# A new prediction model for giant cell arteritis in patients with new onset headache and/or visual loss

**DOI:** 10.1080/07853890.2022.2130971

**Published:** 2022-10-21

**Authors:** Walid Moudrous, Leo H. Visser, Tansel Yilmaz, Marjan H. Wieringa, Tim Alleman, Jörgen Rovers, Mark P.W.A. Houben, Paula M. Janssen, Johan J. B. Janssen, Pieter L. Rensma, Geert J. F. Brekelmans

**Affiliations:** a Department of Neurology, Maasstad Hospital, Rotterdam, the Netherlands; b Department of Neurology, Department of Neurology, Erasmus MC University Medical Center, Rotterdam, the Netherlands; c Department of Neurology of the ETZ, St. Elisabeth Hospital, Tilburg, the Netherlands; d Department of Education and Research, ETZ, Tilburg, the Netherlands; e Department of Neurology, St. Jans Gasthuis Hospital, Weert, the Netherlands; f Department of Neurology, the Gelderse Vallei Hospital, Ede, the Netherlands; g Department of Neurology, the Zuyderland Medical Center, Heerlen, the Netherlands; h Department of Internal Medicine, the Canisius Wilhelmina Hospital, Nijmegen, the Netherlands; i Department of Internal Medicine of the ETZ, location St. Elisabeth Hospital, Tilburg, the Netherlands

**Keywords:** Headache, ultrasound, temporal artery, vasculitis, visual loss, biopsy

## Abstract

**Objective:**

The gold standard for diagnosis of giant cell arteritis (GCA) is a temporal artery biopsy (TAB). We sought for a clinical useful model to predict when an invasive TAB is not necessary to confirm GCA.

**Methods:**

A prospective cohort study was conducted with patients > 50 years with possible GCA, presenting with newly onset headache and/or visual loss. Demographical, clinical, laboratory findings and histological data were collected.

**Results:**

Fifty-six (70%) of the 94 patients showed 1 or more halos of the superficial temporal artery branches. Ultrasound-guided biopsy was positive in 28 patients (30%). Four independent variables predicted a positive TAB: weight loss, bilateral headache, positive halo sign and thrombocytosis. The ROC of the model had an area under the curve of 0.932 with a PPV of 83% and a NPV of 94%.

**Conclusions:**

Weight loss, bilateral headache, a positive halo sign with duplex and thrombocytosis are the most important clinical and laboratory predictors for GCA in a selected group of patients.

**Significance:**

In patients > 50 years presenting with new onset headache or visual loss with 3 or more of the above mentioned risk factors, a biopsy of the temporal artery is not needed to confirm the diagnosis GCA.

**KEY MESSAGES**
- In our study biopsy of the temporal artery was positive in 30% of the patients with possible GCA
- Weight loss, bilateral headache, a positive halo sign on duplex and thrombocytosis are predictors for GCA
- The halo sign had a high sensitivity but a low specificity for a biopsy proven GCA

## Introduction

Giant cell arteritis (GCA) is a vasculitis of the medium- and large-sized arteries, of which the extra-cranial branches of the aorta are preferentially involved [1,2] The prevalence of GCA in the Western world is estimated 20/100.000 in patients older than 50 years and it is the most common primary vasculitis in the elderly [3–5].

Patients may present with a wide range of clinical symptoms; the classical symptoms being new onset headache predominantly in the temporal region, scalp tenderness, fatigue, jaw claudication and bilateral muscle pain in neck, shoulders and pelvic girdles with morning stiffness [1,6]. In addition, visual complaints and fever are frequently reported symptoms. The complications of GCA can be severe and can result in permanent visual loss and neurological deficits caused by ischaemic stroke [7].

For the diagnosis of GCA, biopsy of the superficial temporal artery (TAB) is still considered the gold standard, but it can be normal in up to 20–40% of the cases [5,8]. Furthermore, in a recent study, the kappa between pathologists was only 0,61 [9]. In 1990, the American College of Rheumatology (ACR) published criteria for GCA [10]. These criteria were originally designed as research criteria and they had reasonable performance [10], but if one applies these criteria in clinical practice the positive predictive value of the ACR criteria is only 29% [11]. To obviate the need for an invasive procedure like a TAB, many investigators have sought for prediction models for GCA based on extensive clinical and laboratory criteria, using also contemporary techniques such as duplex scanning. This helped in detecting GCA earlier, but some of these studies were retrospective [12,13].

The aim of our prospective study was to investigate which factors or combination of factors, including clinical characteristics, laboratory tests and duplex imaging characteristics of the temporal artery can predict a positive temporal artery biopsy. We studied this in patients older than 50 years presenting with a new onset headache and/or transient or permanent visual loss in whom neurologists or internists at the outpatient clinic or emergency department suspected a GCA.

## Methods

### Standard protocol approvals, registrations, and patient consents

The Brabant Regional Ethics Committee (NL40526.008.12) and the local committees of the two participating hospitals approved this study. All participants gave written informed consent. We prospectively and consecutively enrolled patients clinically suspected of GCA in two large peripheral teaching centres.

Consecutive patients aged 50 years or older with a new onset headache and/or transient of permanent visual loss who presented to neurologists or internists at the outpatient clinic or emergency department. In some patients a direct cause was found, such as a cerebrovascular disease, infectious cause or a tumour. Patients with new onset headache and/or transient of permanent visual loss in whom no other neurological or internal diagnosis was determined, were included. Exclusion criteria were: (1) a clear indication of an alternative diagnosis explaining the headache or visual loss, such as abnormal clinical findings on physical, ophthalmological or neurological examinations (e.g. hemiparesis, glaucoma, non-arteritic AION [14]); (2) inability to give informed consent; (3) known malignancies, or vasculitis; (4) prior treatment with corticosteroids, and (5) refusal to undergo a temporal artery biopsy.

In all included patients, a thorough patient history and structured clinical examination were performed. When headache was present, the location of the pain was assessed: uni- or bilaterally in the temporal, frontal, parietal or occipital area. We specifically assessed the presence of general malaise, fatigue, weight loss (noting the amount and period of weight loss), fever, muscle pain at the shoulders and upper arms, presence of jaw claudication, diplopia, transient or permanent loss of vision. Patients could also indicate if other symptoms were present. The clinical examination included a general physical and neurological examination, including assessment of the superficial temporal artery (swelling, tenderness and/or redness), and a visual acuity measurement. Additional laboratory data were prospectively collected, including erythrocyte sedimentation rate, C-reactive protein and platelet count. An elevated ESR was defined as a value above the upper limit according to the formula of Miller, [15] an elevated platelet count as >400*10^9^/L, and an elevated CRP as >10 mg/L.

At baseline, all patients underwent an ultrasound examination of the superficial temporal artery. Simultaneous colour Doppler and duplex sonography were performed with a Xario XG ultrasound device (Toshiba, Tokyo, Japan) with a 7–12 MHz linear-array transducer (PLT-1204BT) at the Elisabeth-Tweesteden Hospital Tilburg and with a Hitachi Aloka Prosound Alpha 7 device with a 4–13 MHz linear-array transducer (UST-5411) at the Zuyderland Medical Centre Heerlen. Both common superficial temporal arteries and the frontal and parietal rami were examined in longitudinal and transverse planes. These examinations were performed by specifically trained and experienced clinical neurophysiology technicians, also trained as vascular technologists, who were blinded to the clinical data. They also assessed whether a “halo” was present, which is the most commonly found abnormality in GCA. A halo occurs due to oedematous vessel wall swelling and is characterised by a circumferential hypoechoic wall thickening with a diameter ranging from 0.3 to 2 mm around the vascular lumen [16]. Compression of halo was performed if present.

The superficial temporal artery biopsy (TAB) was performed by a vascular surgeon, and a biopsy was taken within five working days after the first clinical assessment. The biopsy was taken at the symptomatic side. When halos were present, the tract of the vessel showing the halo was marked on the skin, so that the surgeon could perform an echo-guided biopsy. TAB was taken unilaterally on the most symptomatic side. The procedure was performed under local anaesthesia. The minimal required biopsy length was 10 mm. All TABs were examined independently by two trained pathologists, who were blinded to the clinical, laboratory and ultrasound findings. Biopsies were assessed for thickening of the intima layer and presence of a lymphohistiocytic cell infiltrate, consisting of T-lymphocytes and macrophages, histiocytes and multinucleate giant cells. The TABs were considered positive if histological findings were in accordance with international diagnostic guidelines of GCA [17].

We performed statistical analysis with IBM SPSS statistical software for biomedical research, version 22.0 and MedCalc Statistical Software version 14.8.1 (MedCalc Software bvba, Ostend, Belgium). Associations between multiple variables were calculated using chi-squared (or Fisher Exact when cell numbers are less than 5) and Mann-Whitney *U* tests. We performed univariate and multivariate logistic regression analysis. Statistical significance was considered to be present if the *p* value was <0.05. An area under the receiver operating curve was made for the predictive criteria. The probabilities of the different combinations of modalities of the final variables of the logistic regression model were calculated.

## Results

We consecutively enrolled 97 patients during the study period. Most patients (89) were included at the ETZ hospital because the start of the study began much later at the Zuyderland Medical Centre. Four patients were excluded because they did not get a temporal artery biopsy. These patients did not give consent for a biopsy or logistic problems prevented to get a temporal biopsy within the predefined period of 5 working days. The obtained superficial temporal artery biopsies had a median length of 11.8 mm (*SD* ± 6.8); in 8 of the 93 patients the length of specimen was not described by the vascular surgeon.

The baseline characteristics and laboratory findings of the patients are described in Table 1. Mean age of the total cohort was 71 years (*SD* ± 9) and there were slightly more women: 49 (53%). The most common complaint was headache in 82 (89%) of the 93 patients, being bilateral in 27. Thirteen (48%) of the 27 patients with bilateral headache had a biopsy proven GCA, while this occurred in 7 (14%) of the 50 patient with left or right sided reported headache (information of localisation missing in 5 patients), *p* = .001). Temporal location of the headache was most common: 26 (32%) patients, followed by frontal location in 18%, frontotemporal in 16%, diffuse in the head in 16%, occipital in 7% and 11% in other areas. Location of the headache did not differ between the biopsy positive and negative group. Twenty-six patients presented with visual loss; 11 patients with permanent visual loss and 15 patients with transient visual loss. Other frequently reported clinical symptoms were fatigue in 45 patients (48%), general malaise in 44 patients (47%) and weight loss in 24 patients (25%). Mean weight loss was 6.3 kg (range 2–15, Interquartile range 3–10 kg). Polymyalgia rheumatica was reported in only 11 patients (12%). The laboratory results showed an elevated ESR in 70 patients (75%), 42 (45%) had an elevated CRP level and in 14 patients (15%) an elevated platelet count was observed. Data on CRP and platelet count were missing in 12 and 16 patients respectively.

**Table 1.** Baseline clinical and laboratory findings of 93 patients suspected of giant cell arteritis.

Main patient characteristics	Total cohort (*N* = 93)
Female gender *N* (%)	49 (52.7)
Age (years ±*SD*)	71 (± 9.1)
Headache *N* (%)	81 (87.1)
Malaise *N* (%)	44 (47.3)
Fatigue *N* (%)	45 (48.4)
Weight loss *N* (%) (kilograms mean ±*SD*)	24 (25.8%), 6.3 kg.(± 3.4)
Fever *N* (%)	5 (5.3)
Polymyalgia rheumatica *N* (%)	11 (11.8)
Jaw claudication *N* (%)	17 (18.3)
Diplopia *N* (%)	11 (11.8)
Transient visual loss *N* (%)	15 ( 16.1)
Permanent visual loss *N* (%)	11 (11.8)
Eye movement disorder *N* (%)	5 (5.3)
Temporal artery examination	
Temporal swelling *N* (%)	13 (13.9)
Temporal redness *N* (%)	2 (2.2)
Temporal pressure pain *N* (%)	42 (45.2)
Absent temporal artery pulsation *N* (%)	18 (19.4)
Positive temporal biopsy *N* (%)	28 (30.1)
Length of temporal artery biopsy (median in mm (25–75 percentile)^†^	10.0 (8.00-14.5)
Positive Halo sign *N* (%)	65 (69.9)
Laboratory findings	
Elevated ESR level for age and sex *N* (%)^‡^	70 (75.3)
Elevated CRP level (CRP > 10 mg/L) *N* (%)^§^	42 (45.2)
Thrombocytosis (platelets > 400*109/L.) *N* (%)	13 (14.0)

Abbreviations: TAB: temporal artery biopsy; ESR: erythrocyte sedimentation rate; CRP: C-reactive protein.

† Biopsy length was missing in *N* = 8.

‡ Elevated ESR according the formula of Miller.

§ CRP and thrombocytosis was missing in *N* = 8 and *N* = 12 respectively.

All 93 patients underwent a colour duplex sonography and in 65 patients (70%) duplex sonography showed 1 or more halos of the superficial temporal artery branches All patients underwent bilateral colour duplex in 27 patients (41.5%) bilateral halo was detected. An ultrasound-guided biopsy was performed in 57 (87.6%) of the 65 patients. Biopsy of the temporal artery was positive in 28 (30%) of the 93 patients. In 5 of the 8 patients without ultrasound-guided biopsy the biopsy was positive, thus in 3 negative. In Table 2, the comparison is given between the clinical and laboratory characteristics of the 28 patients with a positive biopsy and the 65 patients with a negative biopsy. A positive halo sign was seen in 26 of the 28 patients (93%) with a positive biopsy versus 39 of the 65 (55%) in the group with a negative TAB (*p*≤.001).

**Table 2.** Patient characteristics of temporal biopsy positive and negative patients and comparative analysis.

Patient characteristics *N* (%)	TAB positive (*N* = 28)	TAB negative (*N* = 65)	*p*-Value
Female gender	16 (57.1)	33 (50.8)	.572
Age (median in years)	74	72	.146
Headache	23 (82.1)	58 (89.2)	.350
Bilateral headache	9 (32.1)	5 (7.7)	.002
Malaise	15 (53.6)	29 (45.3)	.466
Fatigue	13 (46.4)	32 (50.0)	.753
Weight loss	18 (64.3)	6 (9.2)	<.001
Fever	4 (14.8)	1 (1.6)	.026
Polymyalgia rheumatica	6 (22.2)	5 (7.8)	.054
Jaw claudication	9 (32.1)	8 (12.3)	.023
Diplopia	3 (10.7)	8 (12.3)	1.000
Visual loss			
transient	3 (10.7)	12 (18.5)	.354
permanent	2 (7.1)	9 (13.8)	.495
Eye movement disorder	1 (3.6)	4 (6.2)	1.000
Temporal artery examination *N* (%)			
Swelling	8 (28.6)	5 (7.7)	.008
Redness	1 (3.6)	1 (1.5)	.514
Pressure pain	12 (42.9)	30 (46.2)	.769
No pulsation	6 (21.4)	12 (18.8)	.766
Median length (mm)	10.0	10.0	.254
Halo present on ultrasound	26 (92.9)	39 (60.0)	.002
Laboratory *N* (%)			
ESR high	27 (96.4)	43 (66.2)	.002
CRP high	25 (89.3)	17 (29.8)	<.001
Thrombocytosis	11 (39.3)	2 (3.8)	<.001

Abbreviations: TAB: temporal artery biopsy; ESR: erythrocyte sedimentation rate; CRP: C-reactive protein.

Predictive factors for a positive biopsy were weight loss (*p*≤.001), bilateral headache (*p* = .007), fever (*p* = .03), jaw claudication (*p* = .048), swelling at the site of the painful superficial temporal artery (*p* = .02), halos on colour duplex sonography (*p* = .003), and laboratory disorders: elevated ESR, CRP and platelet count (*p* = .004, *p*≤.001 and *p*≤.001 respectively).

**Figure 1.**
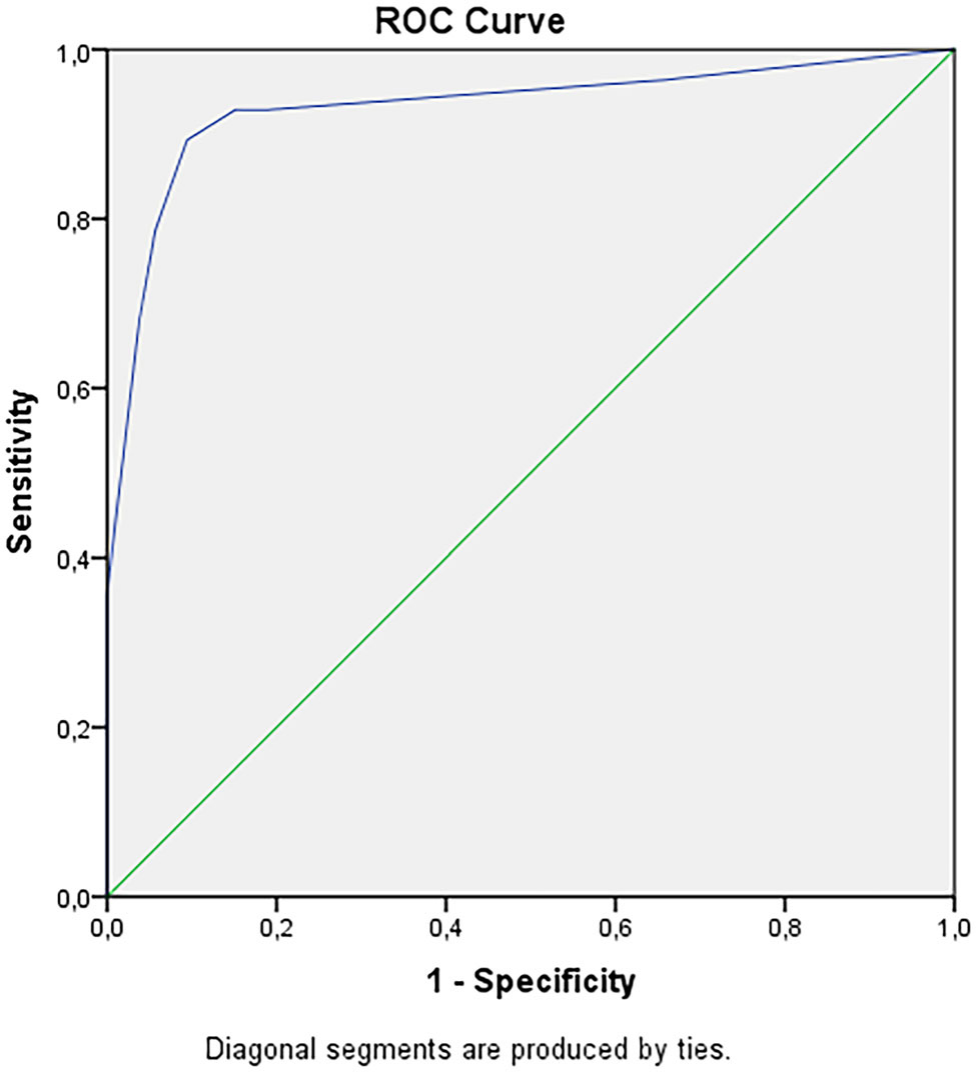
Receiver operating characteristic curve, area =0.932 using the results of the data of Table 3.

A multivariate logistic regression model was made in order to predict a positive TAB in patients suspected of GCA, and the final model consisted of 4 independent variables; weight loss (OR 53; 95% CI 8.2–342), positive halo sign (OR 7.0; 95% CI 1.04–47), thrombocytosis (OR 34; 95% CI 3.4–340) and bilateral headache (OR 20; 95% CI 2.3–167). (Table 3). The receiver operating curve (ROC) was calculated with an area under the curve of 0.932, with a sensitivity of 89% and a specificity of 91% (Figure 1). The positive predictive value was 83% and negative predictive value 94%. With the presence of only 1 of these 4 factors the chance of a positive TAB was less than 35%; when 3 or 4 factors were present the chance of a positive biopsy increased to 97.9 − 99.9%. Chances of a positive TAB varied per combination of 2 present factors: weight loss and thrombocytosis 94.7%, weight loss and bilateral headache 91.2%, weight loss and halo 78.8%, thrombocytosis with bilateral headache 87.1%, halo and thrombocytosis 70.4%, and halo and bilateral headache 58.1%.

**Table 3.** Multiple logistic regression model predicting positive temporal artery biopsy.

Variable	OR (95 % CI)	*p*-Value	AUC
Weight loss	52.9 (8.17–342)	<.001	
Positive halo sign	6.98 (1.04–46.8)	.045	
Thrombocytosis	33.9 (3.39–340)	.003	
Bilateral headache	19.7 (2.32–167)	.006	
			0.932

Abbreviations: AUC: area under the curve.

## Discussion

In this prospective study with specific inclusion criteria, we found that 28 of the 93 (30%) patients older than 50 years and presenting with newly onset of headache and/or visual loss and in whom a GCA was suspected had histologically proven GCA. Multivariate analyses showed that the clinical factors weight loss and bilateral headache, the laboratory finding of an elevated platelet count and the finding of a positive halo on a duplex of the temporal artery were the most predictive for biopsy proven GCA. Our new prediction model using these four factors had a sensitivity of 89% and specificity of 91%, with an area under the ROC curve of 0.932.

Our model gives clinicians and patients equipment for the decision whether a superficial temporal artery biopsy is necessary to confirm the diagnosis of GCA. From a patient perspective, this is an important model because patients are often reluctant to undergo a biopsy of this artery. Disadvantages of taking a biopsy are the need of a length of at least 10 mm, other arteries can be involved and are not biopsied, the low sensitivity (39%) and low kappa (0,61). Earlier studies showed that a greater length of the biopsy increases the chance to detect GCA, but this could not be confirmed in our study. Another advantage of refraining from a biopsy is that it allows follow-up duplex studies of the most affected branch of the temporal artery. A few studies have shown that the disappearance of an initially present halo is a sign of treatment response [16,18].

We have to emphasize that the inclusion criteria in this study were very different from earlier studies on GCA. Most studies included patients who fulfilled the ACR criteria, while we started from a clinical point of view, that is only including patients older than 50 years with new onset headache and/or visual loss with GCA in the differential diagnosis. Patients presenting with a clear indication of an alternative diagnosis explaining the headache or visual loss, such as abnormal clinical findings on physical, ophthalmological or neurological examinations (e.g. hemiparesis, glaucoma, non-arteritic AION) were excluded. The remaining patients could be at risk for diagnosis of GCA and were further assessed following our protocol. Therefore, our data are difficult to compare with their studies. Moreover, this may explain that only 30% had histological proven GCA.

GCA should be diagnosed as soon as possible to prevent (further) visual loss or other complications. Our experience is that the diagnostic process in daily practice is not always easy. A good logistic process is needed, patients may not give consent for a biopsy, and sometimes patients already have been treated with corticosteroids prior to a definite diagnosis. Therefore, this study was undertaken to assess the predictive factors for a positive GCA and to assess whether it was possible to make a predictive model which allows skipping the diagnostic procedure of a TAB in certain patients.

This study shows that when 3 or more of the previously mentioned risk factors are present, a biopsy of the temporal artery is not needed. In recent years fast-track clinics have been developed to improve early diagnosis and treatment of GCA [16]. The implementations of these GCA fast-track clinics has led to a decrease of patients with permanent loss of vision [16]. Our model is a useful instrument to further support the care pathways used in GCA fast-track clinics. All risk factors included in our model can be assessed within one working day, so enabling the diagnosis and treatment of GCA within 24 h.

Our study revealed several important predictive factors not included in the ACR criteria. Of the 97 patients, 87 fulfilled the ACR criteria. Weight loss, bilateral headache, a positive halo sign on duplex and thrombocytosis were the most important predictive factors in our study; all of which are not included in the ACR criteria. Bilateral headache has not been reported as a predictive sign of GCA before, as earlier studies did not differentiate headache types. Thrombocytosis has been reported to be a core sign of GCA, but has not been added yet to the ACR criteria.

Interestingly, only 11% of patients in our study had polymyalgia rheumatica, which is lower than previously reported. This is probably because most patients in this study were referred for work-up to neurologists contrary to patients with polymyalgia rheumatica who are usually referred to rheumatologists by the Dutch general practitioners.

A positive halo sign on duplex can be a sign of GCA, but the reported sensitivity and specificity is greatly variable between studies, with sensitivity ranging from 50–100% and specificity 78–100% [12,16]. In this study we found a high sensitivity but a low specificity despite ultrasound guided biopsies in 87.6% of the patients with a positive halo. Many patients had a clear halo of the superficial temporal artery, but ultrasound-guided biopsy taken at these particular areas did not show inflammation of the temporal artery in 39 of 65 (60%). We did not perform a compression test as suggested by another study [19] and this may have led to false positive findings, but more research is necessary on this subject. In malignant, infectious diseases and in arteriosclerosis of the cranial vessels false-positive halos have been described. There is some debate about the definition of the halo sign, some researchers suggest that intima-media thickness measurement may be more correct for evaluating vasculitic wall oedema than morphologic criteria with colour duplex. But the studies performed are limited and small, and difficult to interpret without a good gold standard like a high positive TAB. Moreover, a discussion has been started about the golden standard in the diagnosis of GCA. The international TABUL study showed that the interobserver agreement on biopsies between pathologists had only a kappa of 0.61 [9]. So, it is possible that in the group with a negative biopsy patients could have GCA. In our study the decision whether a biopsy was negative or positive was decided independently by two trained pathologists.

The strength of this clearly defined cohort study is the prospective design and thorough assessment of clinical symptoms, laboratory tests and duplex sonographic data. All patients suspected of GCA underwent a temporal artery biopsy. However, our study also had several limitations. The main limitation of the study is the relative small number of patients, which is caused by the relative low incidence of GCA, and this is probably the reason for the wide confidence intervals of our findings. Another limitation were the protocol violations: in some patients the minimum required length of the temporal artery biopsy of 10 mm was not obtained and CRP and platelet count were missing in 13% and 17% of the patients. Due to relative small sample size and limited protocol violations could impact the internal validity negatively. In addition, we have not validated our model yet, and this has to be done in a new prospective study with a larger patient cohort. Secondly a meta-analysis to compare current diagnostic models and our proposed model is also much needed to compare difference in diagnostic reliability of these models.

In conclusion, the strongest predictive factors for a positive TAB are weight loss, bilateral headache, a positive halo sign on duplex and thrombocytosis in patients older than 50 years presenting with new onset headache or vision loss. A risk model based on these variables indicates that in patients with 3 or all 4 of these risk factors, a biopsy can be omitted to make the diagnosis of GCA.

## Author contributions

Leo H Visser; Study concept and design, acquisition of data, drafting the manuscript, study supervision.

Walid Moudrous; Study concept and design, acquisition of data, statistical analysis, drafting the manuscript, study supervision.

Tansel Yilmaz; Acquisition of data, study concept and design, acquisition of data, statistical analysis, revising manusscript

Marjan H. Wieringa; Statistical analysis, drafting manuscript, study concept and design, revising manusscript

Tim Alleman; Acquisition of data and interpretation of data, revising manusscript

Jörgen Rovers; Acquisition of data and interpretation of data, revising manusscript

Mark P.W. A. Houben Acquisition of data and interpretation of data, revising manusscript

Paula P. Jansen Acquisition of data and interpretation of data, revising manusscript

Johan J. B. Janssen; Acquisition of data and interpretation of data, revising manusscript

Wiek P.L. Rensma; Study supervision, acquisition of data, revising manusscript

Geert J. F. Brekelmans Study concept and design, acquisition of data, study supervision, revising manusscript

## Ethical statement

The Brabant Regional Ethics Committee (NL40526.008.12) and the local committees of the two participating hospitals approved this study. All participants gave written informed consent.

## Data Availability

Our collected data are available on request, due to privacy legislation.

## Abbreviations

ACR: The American College of Rheumatology
CI: Confidence interval
CRP: C-reactive protein
ESR: Erythrocyte sedimentation rate
GCA: Giant cell arteritis
IQR: Inter Quartile Range
OR: Odds ratio
SD: Standard deviation
TAB: Temporal artery biopsy
